# Translational control of SOG1 expression in response to replication stress in Arabidopsis

**DOI:** 10.1007/s44154-023-00112-2

**Published:** 2023-07-27

**Authors:** Jinchao Li, Weiqiang Qian

**Affiliations:** School of Advanced Agricultural Sciences, Peking University, Beijing, 100871 China

**Keywords:** DNA damage response, ATR, WEE1, SOG1, Translation

## Abstract

DNA damage, which may arise from cellular activities or be induced by genotoxic stresses, can cause genome instability and significantly affect plant growth and productivity. In response to genotoxic stresses, plants activate the cellular DNA damage response (DDR) to sense the stresses and activate downstream processes. The transcription factor SUPPRESSOR OF GAMMA RESPONSE 1 (SOG1), a functional counterpart of mammalian p53, is a master regulator of the DDR in plants. It is activated by various types of DNA lesions and can activate the transcription of hundreds of genes to trigger downstream processes, including cell cycle arrest, DNA repair, endoreplication, and apoptosis. Since SOG1 plays a crucial role in DDR, the activity of SOG1 must be tightly regulated. A recent study published in Plant Cell (Chen et al., Plant Cell koad126, 2023) reports a novel mechanism by which the ATR-WEE1 kinase module promotes *SOG1* translation to fine-tune replication stress response.

Maintaining DNA integrity is crucial for organism survival. However, DNA is constantly attacked by exogenous and endogenous toxic factors, leading to DNA damage and mutations. Plants and animals have evolved a conserved signaling network known as the DNA damage response (DDR), which can sense different types of DNA damage and either arrest the cell cycle to facilitate DNA repair or induce apoptosis to eliminate cells with excessive DNA damage (Yoshiyama 2016). Ataxia-telangiectasia mutated (ATM) and ATM- and RAD3-related (ATR) are two protein kinases that are activated by double-strand DNA breaks (DSBs) and single-strand DNA breaks (SSBs), respectively (Yoshiyama 2016). In mammals, activated ATM or ATR phosphorylates the transcription factor p53, which can trigger a transcriptional cascade to facilitate a broad spectrum of cellular responses (Oren and Rotter 2010). However, plants lack a p53 homolog. A forward genetic screening identified a gene locus called *SUPPRESSOR OF GAMMA RESPONSE 1* (*SOG1*) as a factor required for DNA damage-induced cell cycle arrest in Arabidopsis (Preuss and Britt 2003). SOG1 is a NAC [petunia *NAM* (no apical meristem) and Arabidopsis *ATAF1,2* and *CUC2*] transcription factor and can activate hundreds of genes upon ionizing radiation (Yoshiyama et al. 2009; Bourbousse et al. 2018; Ogita et al. 2018). SOG1 can be phosphorylated and activated by ATM and ATR (Yoshiyama et al. 2013; Sjogren et al. 2015). SOG1 has been found to coordinate the DDR triggered by DSBs, SSBs, replication stress, and many other genotoxic stresses (Adachi et al. 2011; Pedroza-García et al. 2017; Li et al. 2022, 2023). SOG1 was considered as a counterpart of p53 in plants, although their sequences are not similar.

Under normal growth conditions, loss of *SOG1* has little effect on the development of Arabidopsis and rice (Li et al. 2022; Nishizawa-Yokoi et al. 2023). Under genotoxic stress, SOG1 mediates cell cycle arrest and programmed cell death (PCD) in the root tip, and root growth of the *sog1* mutant is less sensitive to genotoxic stress compared to wild-type plants (Sjogren et al. 2015). When genotoxic stress withdraws, SOG1 plays a role in guiding root meristem regeneration and restarting root growth. The *sog1* mutant fails to recover root growth when genotoxic stress withdraws (Johnson et al. 2018). When seeds germinated with excessive DNA damage during storage in nature, SOG1 protects the mitotic competence of meristematic cells in the radicle (Li et al. 2022).

The activity of SOG1 must be tightly controlled to ensure that SOG1 is inactivated in the absence of DNA damage but quickly activated upon DNA damage. In addition to being phosphorylated by ATM, ATR, and CK2 (Yoshiyama 2016; Wei et al. 2021), SOG1 protein can be ubiquitinated by the plant-specific ubiquitin E3 ligase DNA DAMAGE RESPONSE MUTANT 1 (DDRM1). Ubiquitination by DDRM1 increases the stability of SOG1 and thus promotes homologous recombination repair (Wang et al. 2022).

The accurate replication of DNA is essential for the successful transmission of genetic information in all organisms during cell divisions. Nevertheless, DNA replication can be disrupted by a variety of elements, such as ultraviolet radiation, limited nucleotides, and DNA damage (Zeman and Cimprich 2014). ATR and the downstream kinase WEE1 are required for the activation of DNA replication stress responses. However, the downstream signaling pathways and molecular mechanisms are still not fully understood. To elucidate the mechanisms of ATR-WEE1-mediated DNA replication stress response, Chen and colleagues performed a forward genetic screening to isolate regulators in the ATR-WEE1 pathway. In this genetic screening system, the *atr* and *wee1* mutants are supersensitive to the DNA damage agent hydroxyurea (HU), which causes replication stress by inhibiting dNTP biosynthesis (Culligan et al. 2004), and show a short-root phenotype when HU is present in the medium. Through screening suppressor of *atr* (*soat*) mutants that have recovered root growth, they identified GENERAL CONTROL NONDEREPRESSIBLE 20 (GCN20), a highly conserved translational inhibitor, as one of key factors that regulate replication stress response.

Biochemical experiments showed that WEE1 could directly interact with GCN20 and phosphorylate it at Tyr313 and Tyr417 residues. Furthermore, WEE1 promotes the polyubiquitination and subsequent degradation of GCN20, which depends on the kinase activity of WEE1. GCN20 cooperates with GCN1 to activate the kinase activity of GCN2, which then phosphorylates EUKARYOTIC TRANSLATION INITIATION FACTOR 2α (eIF2α) to inhibit protein translation (Izquierdo et al. 2018). In mammalian cells, GCN20 can repress the translation of p53 (Zhou 2011). Interestingly, Chen and colleagues showed that GCN20 could also repress the translation of *SOG1* in Arabidopsis, suggesting that GCN20 functions upstream of SOG1. To further demonstrate this, Chen and colleagues created *SOG1* mutations in the *wee1 gcn20* double mutant background and overexpressed *SOG1* in the *atr* and *wee1* mutants. The loss of *SOG1* abolished the resistance of *wee1 gcn20* to replication stress, whereas overexpressing *SOG1* enhanced the resistance to *atr* or *wee1* to replication stress.

In summary, Chen and colleagues discovered a novel regulatory mechanism for SOG1-mediated signaling under genotoxic stress (Fig. 1). They found that increasing the translation of SOG1 is an important step in ATR-WEE1-mediated DNA replication stress responses. Under DNA replication stress, ATR activates its downstream protein kinase WEE1 to phosphorylate GCN20, a protein involved in translation inhibition. Phosphorylated GCN20 is targeted for polyubiquitination and degradation, allowing translational derepression of *SOG1* mRNAs (Chen et al. 2023). By establishing a connection between protein translational regulation and DNA replication stress, this study significantly advances our understanding of the signal transduction in plant DDR and the molecular mechanisms regulating SOG1 abundance.

**Fig. 1:**
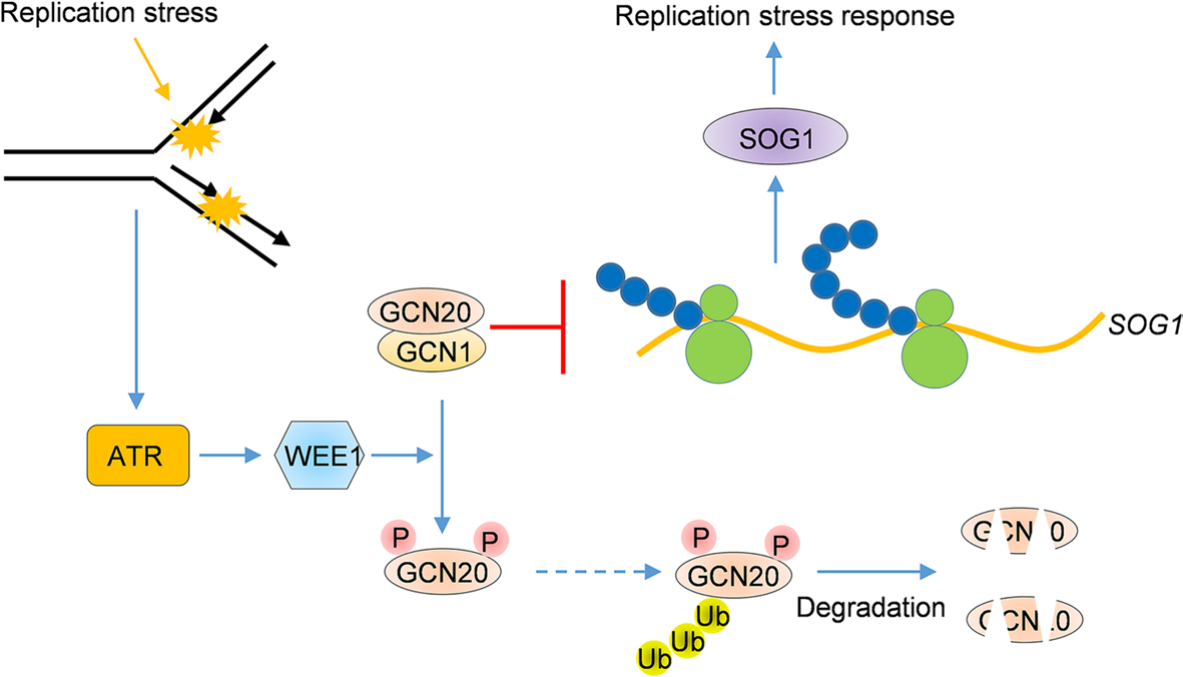
Regulation replication stress response at the translational level. The ATR-WEE1 module is triggered by DNA replication stress, leading to the phosphorylation of GCN20 and consequent polyubiquitination and degradation, thus relieving the inhibition of *SOG1* translation

## Data Availability

Not applicable.

## Abbreviations

DDR: DNA damage response
SOG1: SUPPRESSOR OF GAMMA RESPONSE 1
DSBs: Double-strand DNA breaks
ATM: Ataxia-telangiectasia mutated
ATR: ATM- and RAD3-related
SSBs: Single-strand DNA breaks
NAC: Petunia *NAM* (no apical meristem) and Arabidopsis *ATAF1,2* and *CUC*]
PCD: Programmed cell death
GCN20: GENERAL CONTROL NONDEREPRESSIBLE 20
DDRM1: DNA DAMAGE RESPONSE MUTANT 1
HU: Hydroxyurea

## References

[CR1] Adachi S, Minamisawa K, Okushima Y, Inagaki S, Yoshiyama K, Kondou Y, Kaminuma E, Kawashima M, Toyoda T, Matsui M, Kurihara D, Matsunaga S, Umeda M (2011). Programmed induction of endoreduplication by DNA double-strand breaks in Arabidopsis. Proc Natl Acad Sci USA.

[CR2] Bourbousse C, Vegesna N, Law JA (2018). SOG1 activator and MYB3R repressors regulate a complex DNA damage network in Arabidopsis. Proc Natl Acad Sci USA.

[CR3] Chen H, Pan T, Zheng X, Huang Y, Wu C, Yang T, Gao S, Wang L, Yan S (2023) The ATR-WEE1 kinase module promotes SUPPRESSOR OF GAMMA RESPONSE 1 translation to activate replication stress responses. Plant Cell koad126. 10.1093/plcell/koad126 10.1093/plcell/koad126 PMC10396359 37159556

[CR4] Culligan K, Tissier A, Britt A (2004). ATR regulates a G2-Phase cell-cycle checkpoint in Arabidopsis thaliana. Plant Cell.

[CR5] Izquierdo Y, Kulasekaran S, Benito P, López B, Marcos R, Cascón T, Hamberg M, Castresana C (2018). Arabidopsis nonresponding to oxylipins locus *NOXY7* encodes a yeast GCN1 homolog that mediates noncanonical translation regulation and stress adaptation. Plant Cell Environ.

[CR6] Johnson RA, Conklin PA, Tjahjadi M, Missirian V, Toal T, Brady SM, Britt AB (2018). SUPPRESSOR OF GAMMA RESPONSE1 Links DNA damage response to organ regeneration. Plant Physiol.

[CR7] Li J, Liang W, Liu Y, Ren Z, Ci D, Chang J, Qian W (2022). The Arabidopsis ATR-SOG1 signaling module regulates pleiotropic developmental adjustments in response to 3'-blocked DNA repair intermediates. Plant Cell.

[CR8] Li J, Wang C, Liang W, Zhang J, Jiang CK, Liu Y, Ren Z, Ci D, Chang J, Han S, Deng XW, Wang Y, Qian W (2023). Functional importance and divergence of plant apurinic/apyrimidinic endonucleases in somatic and meiotic DNA repair. Plant Cell.

[CR9] Nishizawa-Yokoi A, Motoyama R, Tanaka T, Mori A, Iida K, Toki S (2023). SUPPRESSOR OF GAMMA RESPONSE 1 plays rice-specific roles in DNA damage response and repair. Plant Physiol.

[CR10] Ogita N, Okushima Y, Tokizawa M, Yamamoto YY, Tanaka M, Seki M, Makita Y, Matsui M, Okamoto-Yoshiyama K, Sakamoto T, Kurata T, Hiruma K, Saijo Y, Takahashi N, Umeda M (2018). Identifying the target genes of SUPPRESSOR OF GAMMA RESPONSE 1, a master transcription factor controlling DNA damage response in Arabidopsis. Plant J.

[CR11] Oren M, Rotter V (2010). Mutant p53 gain-of-function in cancer. Cold Spring Harb Perspect Biol.

[CR12] Pedroza-García JA, Mazubert C, Del Olmo I, Bourge M, Domenichini S, Bounon R, Tariq Z, Delannoy E, Piñeiro M, Jarillo JA, Bergounioux C, Benhamed M, Raynaud C (2017). Function of the plant DNA polymerase Epsilon in replicative stress sensing, a genetic analysis. Plant Physiol.

[CR13] Preuss SB, Britt AB (2003). A DNA-damage-induced cell cycle checkpoint in Arabidopsis. Genetics.

[CR14] Sjogren CA, Bolaris SC, Larsen PB (2015). Aluminum-dependent terminal differentiation of the Arabidopsis root tip is mediated through an ATR-, ALT2-, and SOG1-regulated transcriptional response. Plant Cell.

[CR15] Wang X, Wang L, Huang Y, Deng Z, Li C, Zhang J, Zheng M, Yan S (2022). A plant-specific module for homologous recombination repair. Proc Natl Acad Sci USA.

[CR16] Wei P, Demulder M, David P, Eekhout T, Yoshiyama KO, Nguyen L, Vercauteren I, Eeckhout D, Galle M, De Jaeger G, Larsen P, Audenaert D, Desnos T, Nussaume L, Loris R, De Veylder L (2021). Arabidopsis casein kinase 2 triggers stem cell exhaustion under Al toxicity and phosphate deficiency through activating the DNA damage response pathway. Plant Cell.

[CR17] Yoshiyama KO (2016). SOG1: a master regulator of the DNA damage response in plants. Genes Genet Syst.

[CR18] Yoshiyama K, Conklin PA, Huefner ND, Britt AB (2009). *Suppressor of gamma response 1 (SOG1*) encodes a putative transcription factor governing multiple responses to DNA damage. Proc Natl Acad Sci USA.

[CR19] Yoshiyama KO, Kobayashi J, Ogita N, Ueda M, Kimura S, Maki H, Umeda M (2013). ATM-mediated phosphorylation of SOG1 is essential for the DNA damage response in Arabidopsis. EMBO Rep.

[CR20] Zeman M, Cimprich K (2014). Causes and consequences of replication stress. Nat Cell Biol.

[CR21] Zhou J (2011). Biological functions of human *PTPN4*, *ABCF3*, and *PINX1* genes [Ph.D. thesis].

